# Protein folding of the SAP domain, a naturally occurring two-helix bundle

**DOI:** 10.1016/j.febslet.2015.06.002

**Published:** 2015-07-08

**Authors:** Charlotte A. Dodson, Eyal Arbely

**Affiliations:** a MRC Centre for Protein Engineering, Hills Road, Cambridge CB2 0QH, UK; b Molecular Medicine, National Heart & Lung Institute, Imperial College London, SAF Building, London SW7 2AZ, UK

**Keywords:** SAP domain, Protein folding, Φ-value, Transition state analysis, Tho1

## Abstract

The SAP domain from the *Saccharomyces cerevisiae* Tho1 protein is comprised of just two helices and a hydrophobic core and is one of the smallest proteins whose folding has been characterised. Φ-value analysis revealed that Tho1 SAP folds through a transition state where helix 1 is the most extensively formed element of secondary structure and flickering native-like core contacts from Leu35 are also present. The contacts that contribute most to native state stability of Tho1 SAP are not formed in the transition state.

## Highlights

• Thol SAP domain is one of the smallest model protein folding domains.
• SAP domain folds through a diffuse transition state in which helix 1 is most formed.
• Native state stability is dominated by contacts formed after the transition state.

## 1 Introduction

The principles which govern the folding and unfolding of proteins have fascinated the scientific community for decades [1]. One of the most successful approaches has been to apply chemical transition state theory and treat the folding reaction as a barrier-limited process between two conformational ensembles of proteins populated at equilibrium: the native and denatured state ensembles.

The transition state, the high energy conformation transiently adopted by the polypeptide chain as the protein crosses the barrier between native and denatured ensembles, provides information on the structural mechanism of protein folding. This information cannot be determined by traditional structural techniques, and instead is inferred from kinetics and mutational analysis using the technique of Φ-value analysis [2–7]. The Φ-value of a mutation is defined as the change in stability of the transition state upon making the mutation $\left(\Delta \Delta G_{‡\text{-}D}=\Delta G_{‡\text{-}D}^{\mathrm{WT}}-\Delta G_{‡\text{-}D}^{\mathrm{mut}}\right)$ expressed as a fraction of the change in stability of the native state $\left(\Delta \Delta G_{N\text{-}D}=\Delta G_{N\text{-}D}^{\mathrm{WT}}-\Delta G_{N\text{-}D}^{\mathrm{mut}}\right)$ for the same mutation: *i.e.* $\Phi =\frac{\Delta \Delta G_{‡\text{-}D}}{\Delta \Delta G_{D\text{-}N}}$. For non-disruptive deletion mutations where reorganisations in native or denatured state structure are not predicted, a value of Φ = 1 indicates that any interactions made by the deleted sidechain or chemical group are as formed in the transition state as they are in the native state. A value of Φ = 0 indicates that the interactions are not present in the transition state, and a fractional value of Φ may indicate partial formation of interactions, complete formation of a subset of multiple interactions, or complete formation of interactions in a fraction of cases (*i.e.* heterogeneity in the ensemble of transition states).

In order to draw general conclusions about the principles governing protein folding and unfolding, it is important to determine detailed folding information (including structural information on the transition state) for a number of model proteins of different sizes, structures and topologies. We have previously presented the folding and unfolding behaviour of the L31W (fluorophore) mutant of the SAP domain from the *Saccharomyces cerevisiae* Thol protein [8] (SAP so-named after the first initial of the three proteins in which it was first identified [9]). Thol SAP is monomeric in solution and folds reversibly in an apparent two-state transition making it ideal for further study. The overall fold comprises just 51 residues, which form two approximately parallel helices separated by an extended loop, and possesses a hydrophobic core of just four residues (Leu13, Leu17, Trp31 and Leu35). Its motif of two parallel helices is quite unusual – model α-helical proteins more frequently contain antiparallel or perpendicular helices in a helix-turn-helix arrangement [10–14] – and Tho1 SAP is one of the smallest proteins whose folding has been studied experimentally. It is therefore of interest to study the folding of Tho1 SAP in more detail.

In this paper we have conducted a Φ-value analysis of the Tho1 SAP domain. The Φ-values we obtained were fractional, indicating that Tho1 SAP folds through a transition state with transient formation of a core and flickering elements of helical structure. The best formed element of secondary structure was helix 1. Interestingly, the contacts which contributed most to native state stability were not formed in the transition state. In order to obtain a crude indication of the validity of our results across multiple temperatures, we measured the folding of L31W SAP across the range of 283–323 K. As judged by the change in solvent-accessible surface area upon folding (*β_T_* value and analogous ratio of heat capacities), there are no gross changes in the transition state of Tho1 SAP with temperature.

## 2 Materials and methods

### 2.1 Reagents

L31W SAP domain was expressed and purified as detailed previously [8]. Point mutations were generated using Stratagene Quikchange mutagenesis. Mutant proteins were expressed and purified as described for SAP L31W until completion of cleavage of the fusion protein, when tag and target were separated by flowing once more through Ni-charged IMAC resin (GE Healthcare BioSciences, Sweden) before concentration and gel filtration on an S75 column into 50 mM MES pH 6.0, total ionic strength of 500 mM made up to this value using NaCl. A single peak was obtained for all proteins and fractions within this peak pooled.

### 2.2 Equilibrium denaturation

Far-UV CD spectroscopy (thermal denaturation) and fluorescence emission (chemical denaturation) were carried out as described previously [8].

### 2.3 Kinetic measurements

We measured relaxation kinetics on the μs–ms timescale using T-jump fluorescence spectroscopy and temperature jumps of 3–5 K on a modified Hi-Tech PTJ-64 (Hi-Tech Ltd., Salisbury, UK) capacitor-discharge T-jump apparatus as previously described [8]. Arrhenius analysis of the plot of microscopic rate constant against temperature was carried out constraining the overall Δ*H*, Δ*S* and Δ*C*_p_ to their equilibrium values at the thermal midpoint. Urea denaturation (chevron) plots were fitted to an apparent two-state transition (Eq. 1) with each point weighted by the fitting error on the rate constant. No further constraints were placed upon the data fit:

(1) $$k_{\text{obs}}=k_{\text{f}}e^{\frac{-m_{‡\text{-}D}\cdot [\text{urea}]}{\mathrm{RT}}}+k_{\text{u}}e^{\frac{-m_{‡\text{-}N}\cdot [\text{urea}]}{\mathrm{RT}}}$$

### 2.4 Estimating Φ-values for severely destabilised mutants

We estimated the group (low, medium or high) into which the Φ-values for L17A, R20A and L35A were likely to fall by using our measured data to place bounds on *k*_f_ for these severely destabilised mutants. The data for all three destabilised mutants describe the unfolding arm of the chevron well (*m*_‡-*N*_ and *k*_u_) and we used the average value of *m*_‡-*D*_ from all other mutants (480 ± 21 cal mol^−1^ M^−1^) as a fixed parameter in fitting these chevrons to enable convergence on a solution. We also fit the data with *m*_‡-_*_D_* fixed at 400 cal mol^−1^ M^−1^ and 600 cal mol^−1^ M^−1^ to check that constraining this value did not overly affect the value of *k*_f_ obtained (Supplementary Table SI). In addition, we fit the data for the R20A mutant using Eq. (1) with all parameters allowed to float freely. In all cases, the value of [*D*]_50%_ calculated from the kinetic data was used in calculating Φ and was checked against the data from equilibrium urea denaturation to ensure that it was consistent with this.

#### 2.4.1 R20A chevron

As an additional check on data fitting, we observed that the measured rate constant for R20A in 0 M urea is at, or is a little above, the midpoint of the transition (visual inspection of Fig. 2d). This places an upper bound of 1500 s^−1^ on *k*_f_ (half the measured observed rate constant), consistent with the values in Supplementary Table SI. As a final check on our estimate of Φ, we recalculated this parameter using a ΔΔ*G_N_*_-_*_D_* of 2.1 kcal mol^−1^. This is the most destabilised transition we have been able to fit (D39N) and represents the minimum value of ΔΔ*G_N_*_-_*_D_* (maximum value of Φ) for this mutant. We are thus confident in an estimate of Φ for R20A as <0.3.

#### 2.4.2 L17A chevron and L35A chevrons

All data fits resulted in a Φ-value of 0.1 for L17A and 0.3 for L35A. We were a little concerned that the data fits indicated that the L35A mutant was less stable than L17A when the chevrons suggest the reverse (urea concentration of maximum measurable signal higher for L17A). In order to determine the impact of this on the Φ-value calculated, we used the fitted values of *k*_f_ to calculate Φ-values with destabilisations corresponding to [*D*]_50%_ values of 1.3 M (ΔΔ*G_N_*_-_*_D_* = 2.1 kcal mol^−1^), 0 M and −2.0 M urea (L35A) and 1.3 M, −2.0 M and −4.0 M urea (L17A). For L17A, using the minimum value of 2.1 kcal mol^−1^ for ΔΔ*G_N_*_-_*_D_* raised Φ to 0.2 for all but the lowest value of *m*_‡-_*_D_*. Other values of [*D*]_50%_ left Φ = 0.1 (unchanged) indicating a likely value of 0.1. For L35A, the maximum value of Φ was 0.5/0.6 (ΔΔ*G_N_*_-_*_D_* = 2.1 kcal mol^−1^) with a value of 0.4 when [*D*]_50%_ = 0 M and unchanged (Φ = 0.3) when [*D*]_50%_ = −2.0 M. Since we estimate the midpoint of this mutant to be between 0 M and −2.0 M, we assign the Φ-value for L35A to the ‘medium’ category.

## 3 Results

### 3.1 Contribution of specific side-chains to native state stability

**Fig. 1:**
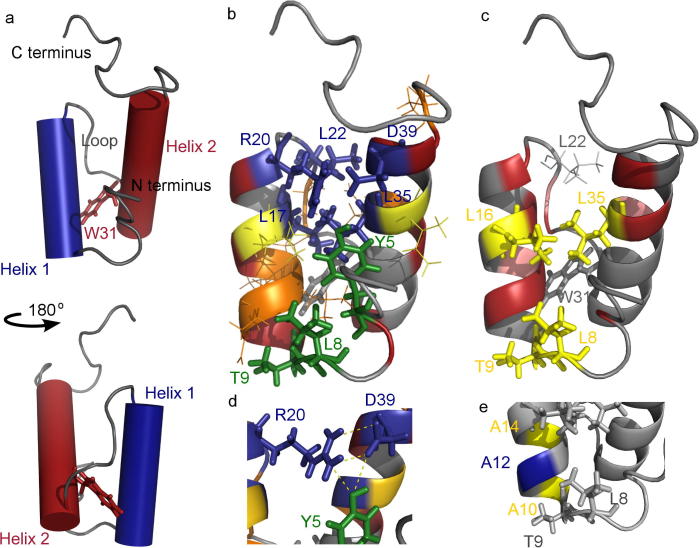
Structure of L31W and energetic contribution of different residues to stability. (a) Connectivity of L31W. Trp31 is shown in stick representation. (b) Contribution of side chains to native state stability. −0.5 < ΔΔG*_N_*_-_*_D_* < 0.5 kcal mol^−1^ (red), 0.5 < ΔΔG*_N_*_-_*_D_* ⩽ 1.0 kcal mol^−1^ (orange), 1.0 < ΔΔG*_N_*_-_*_D_* ⩽ 1.5 kcal mol^−1^ (yellow), 1.5 < ΔΔG*_N_*_-_*_D_* ⩽ 2.0 kcal mol^−1^ (green), ΔΔG*_N_*_-_*_D_* > 2.0 kcal mol^−1^ (blue). Trp31 is shown in grey. (c) Contribution of side chains to transition state stability. Φ-Values coloured by Φ_f_ < 0 (pink), 0 ⩽ Φ_f_ < 0.3 (red), 0.3 ⩽ Φ_f_ ⩽ 0.6 (yellow) and Φ_f_ > 0.6 (blue). Trp31 and Leu22 shown in grey. (d) Putative hydrogen bonding network stabilising the native state of Tho1 SAP. Proposed hydrogen bonds shown with dashed yellow lines, other colours as for (b). (e) Φ-values for alanine-glycine scanning of helix 1 with A10G, A12G and A14G. Colours as for (c).

As a preparation for Φ-value analysis, and in order to probe the contribution of different residues to the native state stability of L31W, we made a series of thirty-five non-disruptive side-chain deletion mutations (Table 1, Fig. 1b and Supplementary Fig. S1). The greatest contribution to L31W stability (>2 kcal mol^−1^) was made by a cluster of residues on the buried faces of the C-terminal end of both helices and at the beginning of the connecting loop. Of the five residues making up this cluster, Leu17 and Leu35 are buried in the hydrophobic core of the SAP domain, and Leu22 and the alkyl chain of Arg20 pack against them. Asp39 and the guanidinium group of Arg20 are solvent-exposed.

To probe the role of the surface-exposed residues further, we mutated Asp39 to both alanine and asparagine. Both mutations destabilised L31W by similar amounts (2.2 and 2.6 kcal mol^−1^) indicating that the carboxylic acid group in the aspartate is forming a stabilising interaction. Mutating Arg20 to alanine destabilised L31W by a greater amount (>2.6 kcal mol^−1^) than mutating Asp39. 2.0–2.5 kcal mol^−1^ of this destabilisation is likely to arise from loss of a hydrogen bond or salt bridge between the guanidinium group of Arg20 and the carboxylic acid of Asp39 (Fig. 1d), and the remaining destabilisation is likely to be due to the loss of the Arg20 alkyl chain packing against the hydrophobic core. We speculate that a further hydrogen bond may be formed between Asp39 and Tyr5. Like Arg20, Tyr5 is close to Asp39 and removal of the tyrosine hydroxyl destabilised L31W considerably (Y5F mutation; 2.4 kcal mol^−1^).

Most residues within helix 1 played little role in stabilising the native state. However, comparison of T9S and T9A mutants enabled us to determine the energetic contribution of the N-capping Thr9 hydroxyl group to the overall stability of L31W (*i.e.* to determine the contribution of the virtual mutation S9A) to be 1.3 kcal mol^−1^. Leu8 acts as a hydrophobic staple (L8A gave a ΔΔ*G_D_*_-_*_N_* of 2.0 kcal mol^−1^).

### 3.2 Φ-value analysis of SAP L31W

**Fig. 2:**
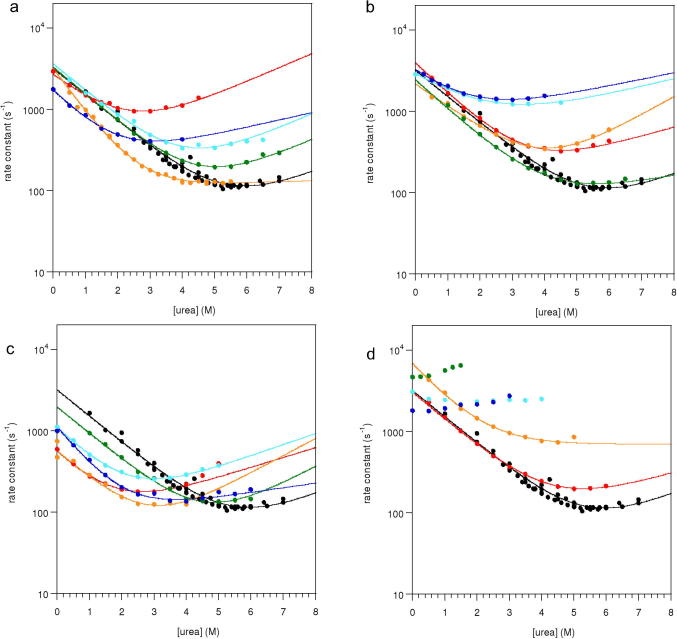
Chevron plots for L31W mutants. (a) *pseudo-wildtype* (black), Y5F (red), Q12A (orange), L13A (green), K14A (light blue), K14G (dark blue). (b) *pseudo-wildtype* (black), S23A (red), I36A (orange), I36V (green), D39A (light blue), D39N (dark blue). (c) *pseudo- wildtype* (black), L8A (red), T9A (orange), V10G (green), Q12G (light blue), L16A (dark blue). (d) *pseudo-wildtype* (black), V24A (red), R34A (orange), L17A (dark green), R20A (light blue), L35A (dark blue). No fit shown for very destabilised mutants.

In order to build a picture of the transition state of the SAP domain, we undertook a Φ-value analysis of L31W. Twenty of our thirty-five point mutants were suitable for Φ-value analysis [15]. All gave rise to apparent two-state chevron plots with linear arms (Fig. 2) which, in most cases, could be fitted with high confidence to yield Φ-values (Table 2). Three mutants, L17A, R20A and L35A, were so destabilised that it was not possible to fit the folding arm of the chevron reliably. However by placing bounds on *k*_f_ and on the equilibrium denaturation midpoint, and by careful analysis of the resulting data fits, we could estimate into which category the Φ-values for these mutants fell (see Section 2.4 for full details).

Most Φ-values measured were fractional, which we interpret to indicate that the transition state ensemble for SAP L31W does not contain extensive regions of fully consolidated secondary structure (Fig. 1c & e). The highest Φ-values (Φ = 0.5–0.6) were measured for Leu8, Thr9 and Leu16. Leu8 and Thr9 form the base of helix 1, while Leu16 (on helix 1) participates in core hydrophobic packing. Alanine to glycine mutations at the N-terminus of helix 1 (which probe the degree of helix formation [2,16]) gave Φ-values ranging from 0.3 to 0.7, although the error on the largest value is considerable. Together, these results indicate that helix 1 is partially formed in the transition state for folding.

Our Φ-value mutants provide several probes of the degree of formation of the hydrophobic core in the L31W transition state. Leu13 and Arg20 both have low Φ-values indicating that their core contacts are not formed in the transition state. Leu35 (helix 2) and Leu16 (helix 1) have medium Φ-values, with that for Leu35 being lower than that for Leu16. It was not possible to measure a Φ-value for Leu22 since the L22A mutant was too destabilised for kinetic studies. Of our probes, only Leu17 and Leu35 are completely buried from solvent water in the native structure, and their differing Φ-values indicate that the hydrophobic core is not formed in the transition state (if the core were formed, both would have similar degrees of native-like solvation and hence similar Φ-values). Nevertheless, the presence of a medium Φ-value for Leu35 indicates that some hydrophobic collapse occurs in this region. We thus conclude that helix 1 is the most structured region in the transition state, with flickering native-like core contacts from Leu16 and Leu35.

### 3.3 Solvent accessibility of the Thol SAP transition state

Our measurements of SAP domain folding (here and in ref [8]) have all been made at 283 K because the baselines of our equilibrium chemical denaturation are best defined at this temperature and our measurements most accurate. However, many protein folding experiments are carried out at higher temperatures and we wanted some assurance that our conclusions were valid across a range of temperatures.

**Fig. 3:**
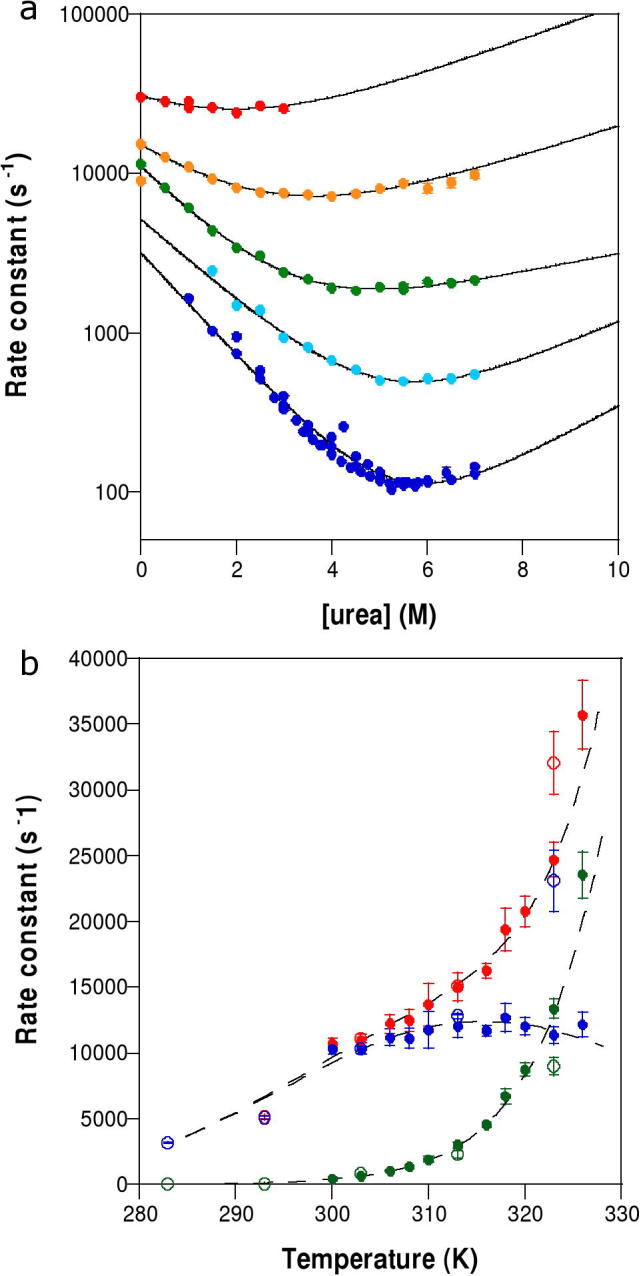
Kinetic behaviour of L31W at different temperatures. (a) Chevron plots for L31W at 283 K (dark blue), 293 K (light blue), 303 K (green), 313 K (orange) and 323 K (red). Unconstrained fit (solid line) and fit constrained by equilibrium [*D*]_50%_ (black dashed line) are shown. (b) Perturbation of L31W folding by temperature. *k*_obs_ shown in red, *k*_f_ in blue and *k*_u_ in green. Solid circles are directly measured in the absence of denaturant at stated temperature, open circles are values extrapolated to absence of denaturant using chevrons in (a). All kinetics measured in 50 mM MES pH 6.0, total ionic strength corrected to 500 mM using NaCl.

The denaturant *m*-value of a protein unfolding transition reports on changes in solvent-accessible surface area (ΔSASA) upon protein denaturation [17]. In order to determine whether the ΔSASA between unfolded and transition states (ΔSASA_‡-_*_D_*) varied with temperature, we measured the kinetics of SAP domain folding at 10 degree intervals between 283 K and 323 K (Fig. 3a and Table 3).

The Tanford *β*-value (*β_T_* = *m*_‡-_*_D_*/*m_D_*_-_*_N_*) reports on ΔSASA_‡-_*_D_* as the fractional ΔSASA upon formation of the transition state. Changes in *β_T_* with temperature or [chemical denaturant] can indicate Hammond behaviour [18–20] or one of many mechanistic changes [21–25]. The average value of *β_T_* for Tho1 SAP was 0.70 ± 0.03 (mean ± S.E.M.) and did not vary with temperature.

We also probed the folding of SAP L31W in buffer alone by measuring the observed rate constant, *k*_obs_, as a function of temperature (Fig. 3b). We included the rate constants in the absence of denaturant from Table 3 as they overlaid the other kinetic data excellently and helped define the curvature in *k*_f_ at low temperatures. The fractional position of the L31W transition state at 322 K (calculated from the ratio Δ*C*_p(‡-_*_D_*_)_/Δ*C*_p(_*_N_*_-_*_D_*_)_ [26]) is also a measure of fractional ΔSASA upon formation of the transition state analogous to *β_T_* [17]. The ratio of heat capacities for SAP L31W was 0.9.

Both *β_T_* and the ratio of heat capacities for Tho1 SAP are high, indicating that the greatest change in solvent accessible surface area occurs between denatured and transition states. Thus the transition state of Tho1 SAP is a compact, partially dehydrated, structured species. The *β_T_* value for Tho1 SAP did not vary with temperature, giving us confidence that the model of the transition state we have determined is likely to be relevant across a range of temperatures.

## 4 Discussion

### 4.1 Brønsted/Leffler analysis of Thol SAP

**Fig. 4:**
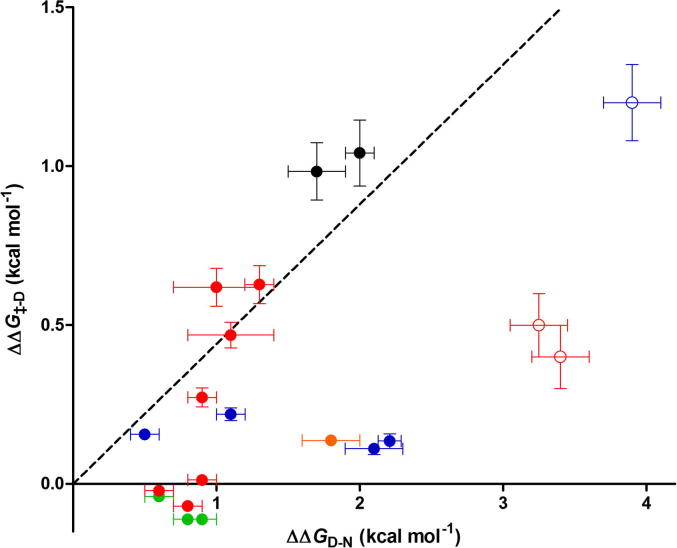
Brønsted plot for Tho1 SAP. Mutations colour-coded by position as Leu8/Thr9 (black), helix 1 (red), connecting loop (green), helix 2 (blue), N-terminus (orange). Dashed line indicates linear regression line for helix 1 and Leu8/Thr9. Filled circles indicate mutants where ΔΔ*G_D_*_-_*_N_* < 2.6 kcal mol^−1^; open circles indicate mutants where ΔΔ*G_D_*_-_*_N_* > 2.6 kcal mol^−1^. Error bars show error from Tables 1 and 2. A minimum error of 10% has been applied to ΔΔ*G*_‡-_*_D_*.

As an alternative method of determining the extent of structure formation in the Tho1 SAP transition state, we plotted ΔΔ*G*_‡-_*_D_* against ΔΔ*G_D_*_-_*_N_* for each of our Φ-value mutants (Fig. 4). By comparison with Brønsted analysis [27] and Leffler *α*-values [28] of organic chemistry, linearity among residues present in the same region of the protein (*e.g.* within an element of secondary structure) indicates that this region is equally formed in the transition state. The gradient of the line gives the extent of formation of this region on a reaction coordinate of free energy [29]. Outliers in this analysis can indicate long-range contacts. Depending on the position of the outlier (*i.e.* above or below the trend line), a point may indicate long-range contacts formed in the transition state, or may indicate contacts formed in the native state but not present in the transition state.

For the SAP domain, there is a weak correlation between ΔΔ*G*_‡-_*_D_* and ΔΔ*G_D_*_-_*_N_* for those residues in helix 1 which make only local contacts in the native state (solid red and black points in Fig. 4; slope of best fit line = 0.4 ± 0.1; *R*^2^ = 0.8). Two points in helix 1 and one point in helix 2 are outliers in this analysis (open circles in Fig. 4). These points are from residues which make long-range contacts in the SAP native state (L17A, R20A and L35A) and deletion of these contacts results in severe destabilisation of SAP domain. The value of ΔΔ*G*_‡-_*_D_* for Leu35 is much higher than expected from the other residues in helix 2. This is consistent with Leu35 (but not the other probes in helix 2) forming native-like long-range contacts in the transition state, and is also indicated by the Φ-values for this residue. Relative to ΔΔ*G*_‡-_*_D_*, ΔΔ*G_N_*_-_*_D_* for both Leu17 and Arg20 is much larger than expected from other residues in helix 1. Both of these residues have low Φ-values, and their position as outliers on the Brønsted plot is consistent with both residues forming long-range contacts in the native state which are not formed in the transition state.

### 4.2 Comparing contacts stabilising the native and transition states

The contacts which contribute most to the native state stability of Tho1 SAP (ΔΔ*G_D_*_-_*_N_* > 2.6) were not formed in the transition state (low Φ-values for Y5F, R20A and D39A/N; and Brønsted analysis above). These contacts form a network of hydrogen bonds in the native state (Fig. 1d). Likewise, the sidechain of Leu17 forms part of the SAP hydrophobic core and contributes >2.6 kcal mol^−1^ to native state stability, yet it only has a Φ-value of 0.1. From this, we conclude that the late stages of Tho1 folding involve ‘locking’ the diffuse transition state ensemble into the native state using strong, long-range interactions.

## 5 Summary & conclusions

We carried out a Φ-value analysis of the folding reaction of the Tho1 SAP domain in order to build a structural model of the transition state. All Φ-values were fractional, indicating that no elements of secondary structure were fully formed in the transition state. Overall, helix 1 was the most highly structured region in the transition state with occasional native-like core contacts from Leu16 and Leu35.

We also probed the folding of the SAP domain by determining the degree of ΔSASA at different temperatures. Both *β_T_* and the analogous ratio of heat capacities indicated a compact transition state. *β_T_* remained constant across the range 283–323 K indicating no gross changes in the folding mechanism.

The contacts which contributed most to Tho1 SAP native state stability were not formed in the transition state. We thus conclude that the early stages of SAP domain folding involve chain collapse and exclusion of solvent water, while the late stages involve formation of strong long-range interactions.

## Figures and Tables

**Table 1:** Equilibrium characterisation of L31W deletion mutants.

Mutation (L31W+)	*T*_*m*_ (K)^a^	Δ*H_D-N_* (at *T*_*m*_) (kcal mol^−1^)^a^	ΔΔ*G_D_*_-_*_N_* at 322 K (kcal mol^−1^)^b^	*m_D_*_-_*_N_* (cal mol^−1^ M^−1^)^c^	[*D*]_50%_ (M)^d^	ΔΔ*G_N-D_* at 4.8 M urea (kcal mol^−1^)^e^
Wild-type	322.4 ± 0.2	33.4 ± 0.4	N/A	690 ± 50	4.8 ± 0.1	N/A

Y5A^f^	N/D	N/D	N/D	N/D	N/D	N/D
Y5F	299	16.8	2.4	620	1.7	1.8 ± 0.2
S6A	323	34.4	−0.1	530	4.6	0.2 ± 0.1

S6G	321	33.4	0.2	930	4.5	0.2 ± 0.1
L8A	303	31.4	2.0	660	1.2	2.0 ± 0.2
T9S	323	35.5	−0.1	520	4.2	0.4 ± 0.1

T9A	N/D	N/D	N/D	670	2.2	1.7 ± 0.2
V10A	321	33.3	0.1	730	4.4	0.3 ± 0.1
V10G	316	33.6	0.7	680	3.5	0.9 ± 0.1

V10A/V11A	326	35.4	−0.3	730	5.0	−0.4 ± 0.1
V11A	326	33.9	−0.4	320	6.9	−0.4 ± 0.1
V11G	323	34.4	−0.1	470	5.1	0.0 ± 0.1

Q12A	317	32.6	0.6	720	3.4	0.9 ± 0.1
Q12G	303	21.1	2.0	730	2.2	1.9 ± 0.2
L13A	317	31.0	0.5	610	3.9	0.6 ± 0.1

K14A	318	31.4	0.4	570	3.3	0.8 ± 0.1
K14G	304	27.5	1.9	690	2.0	1.9 ± 0.2
D15A	325	34.1	−0.3	670	5.0	−0.2 ± 0.1

L16A	310	29.8	1.3	760	2.9	1.3 ± 0.1
L17A^g^	N/D	N/D	N/D	N/D	N/D	N/D
K19A	319	36.5	0.3	380	4.6	0.4 ± 0.1

R20A^g^	N/D	N/D	N/D	N/D	N/D	N/D
L22A^g^	N/D	N/D	N/D	N/D	N/D	N/D
S23A	317	31.9	0.6	590	3.3	0.9 ± 0.1

V24A	317	29.9	0.5	N/D	N/D	N/D
K28A	316	29.3	0.6	550	4.8	−0.0 ± 0.1
Q33A	323	35.3	−0.1	600	5.0	−0.1 ± 0.1

R34A	315	24.0	0.8	570	3.4	0.8 ± 0.1
L35A^g^	N/D	N/D	N/D	N/D	N/D	N/D
I36A	314	29.7	0.9	590	3.0	1.1 ± 0.1

I36V	320	33.5	0.3	660	4.0	0.5 ± 0.1
D38A	323	30.6	−0.1	610	4.2	0.4 ± 0.1
D39A	301	13.7	2.2	N/D	N/D	N/D

D39N	298	13.3	2.6	610	1.3	2.1 ± 0.2
E40Q	322	33.3	0.1	660	4.6	0.1 ± 0.1
E41G	319	31.5	0.4	660	4.1	0.5 ± 0.1

N/A not applicable, N/D not determined.

a S.E.M. reported for wild-type (*n* = 3). Standard deviation on repeat measurements on wild-type and all fitting errors on mutants <1 K for *T*_*m*_ and <0.6 kcal mol^−1^ for Δ*H*.

b ΔΔ*G_D_*_-_*_N_* calculated using the Schellman equation [30]: ΔΔ*G* = (*T*_*m*_*^WT^* − *T*_*m*_^mutant^)$\Delta H_{T_{m}}^{\mathrm{WT}}/T_{m}^{\mathrm{WT}}$. All reported values have a propagated error of 0.1 kcal mol^−1^ (propagation using error of 1 K for *T*_*m*_ of mutant, and S.E.M. for wild-type parameters).

c S.E.M. reported for wild-type (*n* = 6). Fitting error on all values reported for mutants ⩽10 cal mol^−1^ M^−1^.

d [*D*]_50%_ obtained using average *m_D_*_-_*_N_* of 645 ± 14 cal mol^−1^ M^−1^ for fits of mutant data. S.E.M. reported for wild-type (*n* = 6), fitting error ⩽0.1 M for all mutants.

e ΔΔ*G* calculated from Δ[*D*]_50%_ and average *m_D_*_-_*_N_*. Error propagation used an error of 0.2 M in [*D*]_50%_ and 120 cal mol^−1^ M^−1^ in *m_D_*_-_*_N_* for mutant proteins. S.E.M. was used for wild-type parameters.

f Poor protein expression.

g Mutation too destabilising to fit and report parameters for thermal or chemical denaturation curves.

**Table 2:** Kinetic characterisation of L31W deletion mutants.^a^

Mutation (L31W+)	*k*_f_ (s^−1^)^b^	−*m*_‡-_*_D_* (cal mol^−1^ M^−1^)	*k*_u_ (s^−1^)^b^	*m*_‡-_*_N_* (cal mol^−1^ M^−1^)	Calc [*D*]_50%_ (M)	*m_D_*_-_*_N_*_(kinetic)_ (cal mol^−1^ M^−1^) c	Φ^b^
Wild-type	3160 ± 30	420 ± 10	8 ± 5	210 ± 10	5.4	630 ± 10	N/A

Y5F	2480 ± 20	400 ± 10	210 ± 20	220 ± 10	2.3	620 ± 20	0.1 ± 0.1
L8A	500 ± 10	520 ± 10	70 ± 10	160 ± 10	1.7	680 ± 10	0.5 ± 0.1
T9A	600 ± 60	440 ± 10	20 ± 10	280 ± 10	2.8	720 ± 10	0.6 ± 0.1

V10G	1950 ± 20	420 ± 10	8 ± 5	270 ± 90	4.5	690 ± 10	0.3 ± 0.1
A10G	N/A	N/A	N/A	N/A	N/A	N/A	0.3 ± 0.1
Q12A	3090 ± 40	710 ± 10	100 ± 10	20 ± 10	2.6	730 ± 10	0.0 ± 0.1

Q12G	1030 ± 10	510 ± 10	70 ± 10	180 ± 10	2.2	690 ± 10	0.3 ± 0.1
A12G	N/A	N/A	N/A	N/A	N/A	N/A	0.7 ± 0.3
L13A	3280 ± 40	430 ± 10	20 ± 10	220 ± 10	4.5	660 ± 10	0.0 ± 0.1

K14A	3580 ± 20	430 ± 10	40 ± 10	220 ± 50	4.0	650 ± 10	−0.1 ± 0.1
K14G	1550 ± 10	540 ± 10	170 ± 10	120 ± 10	1.9	660 ± 10	0.2 ± 0.1
A14G	N/A	N/A	N/A	N/A	N/A	N/A	0.4 ± 0.2

L16A	1040 ± 10	650 ± 10	80 ± 10	80 ± 10	2.0	720 ± 10	0.5 ± 0.1
L17A	N/D	N/D	N/D	N/D	N/D	N/D	∼0.1^d^
R20A	N/D	N/D	N/D	N/D	N/D	N/D	<0.3^d^

S23A	3850 ± 60	490 ± 10	80 ± 10	150 ± 10	3.4	640 ± 10	−0.1 ± 0.1
V24A	3000 ± 40	430 ± 10	40 ± 10	150 ± 10	4.3	580 ± 10	0.1 ± 0.1
R34A	6170 ± 270	590 ± 40	670 ± 130	3 ± 20	2.1	590 ± 50	−0.5 ± 0.1

L35A	N/D	N/D	N/D	N/D	N/D	N/D	∼0.3–0.6^d^
I36A	2140 ± 20	360 ± 10	20 ± 10	290 ± 10	3.8	650 ± 10	0.2 ± 0.1
I36V	2390 ± 40	470 ± 10	40 ± 10	100 ± 10	4.0	570 ± 10	0.3 ± 0.1

D39A	2490 ± 100	340 ± 30	430 ± 110	120 ± 30	2.1	470 ± 40	0.1 ± 0.1^e^
D39 N	2600 ± 80	440 ± 30	660 ± 100	110 ± 20	1.4	550 ± 40	0.1 ± 0.1

N/A not applicable, N/D not determined.

a Errors quoted are fitting errors. A minimum error of 10 is applied to all fitting errors with the exception of values of *k*_u_ less than 10, where a minimum error of 5 is applied.

b Calculated at 0 M urea. $\Phi =\mathrm{RT}\;\text{ln}\left(\frac{k_{\text{f}}^{L31W}}{k_{\text{f}}^{\mathrm{mut}}}\right)/\Delta {\left[D\right]}_{50\%}\cdot m_{D-N(\mathrm{average})}$. Minimum error of 0.1 reported on Φ.

c *m_D_*_-_*_N_*_(kinetic) = _*m*_‡-_*_N_* − *m*_‡-_*_D_*.

d Kinetic parameters for detailed fits of these very destabilised mutants given in Supplementary Table SI.

e Φ for D39A calculated using average equilibrium *m*-value and estimated [*D*]_50%_.

**Table 3:** Fitted parameters from chevrons at different temperatures.^a^

	283 K	293 K	303 K	313 K	323 K^b^
−*m*_‡-_*_D_* (cal mol^−1^ M^−1^)	420 ± 10	350 ± 10	430 ± 10	300 ± 10	250 ± 20
*m*_‡-_*_N_* (cal mol^−1^ M^−1^)	210 ± 10	170 ± 10	80 ± 10	130 ± 10	160 ± 20
*k*_f_ (s^−1^)	3160 ± 30	5080 ± 140	10 280 ± 80	12 800 ± 220	23 100 ± 2300
*k*_u_ (s^−1^)	8 ± 5	60 ± 10	810 ± 40	2300 ± 250	8950 ± 660
*m_D_*_-_*_N_*_(kinetic)_ (cal mol^−1^ M^−1^)^c^	630 ± 10	520 ± 10	510 ± 10	430 ± 10	410 ± 30
[*D*]_50%(kinetic)_ (M)	5.3 ± 0.6	4.8 ± 0.2	2.8 ± 0.1	2.2 ± 0.2	N/A
*m_D_*_-_*_N_*_(eqm)_ (cal mol^−1^ M^−1^)^d^	690	590	590	N/D	N/D
[*D*]_50%(eqm)_ (M)^e^	4.8 ± 0.1	3.7 ± 0.1	2.6 ± 0.1	2.4 ± 0.1	1.3 ± 0.2
*β_T_*^f^	0.7	0.7	0.8	0.7	0.6

a Errors are fitting errors (or errors propagated from these).

b Chevron at 323 K fit constraining midpoint of chevron to that determined by equilibrium chemical denaturation. Data did not constrain fit in absence of this constraint. Fitting equation was formulated as $k_{\text{obs}}=k_{50}e^{\frac{m_{‡-D}\cdot \left([\text{urea}]-{\left[D\right]}_{50\%}\right)}{\mathrm{RT}}}+k_{50}e^{\frac{m_{‡-N}\cdot \left([\text{urea}]-{\left[D\right]}_{50\%}\right)}{\mathrm{RT}}}$ for this fit. Reported values for *k*_f_ and *k*_u_ are calculated from *k*_50_ (the microscopic rate constant at the denaturation midpoint).

c *m_D-N_*_(kinetic)_ = *m*_‡-*N*_ − *m*_‡-*D*_.

d *m_D_*_-_*_N_*_(eqm)_ is determined from equilibrium chemical denaturation. Fitting error <10 cal mol^−1^ M^−1^, and standard deviation of repeat measurements at 283 K is 100 cal mol^−1^ M^−1^ (*n* = 6).

e [*D*]_50%(eqm)_ determined by equilibrium chemical denaturation (283 K, 293 K, 303 K) or estimated by calculating Δ*G_D_*_-_*_N_* from thermal denaturation and dividing by *m_D_*_-_*_N_* (313 K, 323 K).

f Calculated as *β_T_* = -*m*_‡-_*_D_*/*m*_*D*-_*_N_*_(kinetic)_. Estimated error of 0.1 on all values.

## Supplementary data

Supplementary data This document contains supplementary information.
