# Supplementary material for: Protein folding of the SAP domain, a naturally occurring two-helix bundle
Source: FEBS Lett. 2015 Jul 8;589(15):1740–7. doi: 10.1016/j.febslet.2015.06.002 (PMC4509717; doi:10.1016/j.febslet.2015.06.002)
Supplement: Supplementary data — This document contains supplementary information. [file mmc1.pdf]

Supplementary table S I: Estimating Φ-values for severely destabilised mutants[a]

Mutation (L31W+)		k_f (s^{-1}) [b]	$-m_{‡\text{-}D}$ (cal mol^{-1} M^{-1})	k_u (s^{-1}) [b]	$m_{‡\text{-}N}$ (cal mol^{-1} M^{-1})	Calc $[D]_{50\%}$ (M) [c]	Φ [b]
L17A	Low	1880 ± 500	400	2780 ± 450	290 ± 60	-0.3	0.1
	Ave	1640 ± 460	480	3020 ± 410	270 ± 50	-0.5	0.1
	High	1380 ± 400	600	3290 ± 360	250 ± 40	-0.6	0.1
R20A	Free	1030 ± 70	830 ± 90	2020 ± 70	30 ± 10	-0.4	0.2
	Low	1530 ± 60	400	1430 ± 40	80 ± 10	0.1	0.1
	Ave	1370 ± 50	480	1610 ± 40	70 ± 10	-0.2	0.2
	High	1210 ± 50	600	1800 ± 30	50 ± 10	-0.3	0.2
L35A	Low	440 ± 40	400	1360 ± 30	130 ± 10	-1.2	0.3
	Ave	380 ±30	480	1420 ± 30	120 ± 10	-1.3	0.3
	High	320 ± 30	600	1480 ± 20	110 ± 10	-1.2	0.3

[a] Errors quoted are fitting errors. A minimum error of 10 is applied to all fitting errors.

[b] Calculated at 0 M urea. $\Phi = RT\ln\left(\frac{k_f^{L31W}}{k_f^{mut}}\right) \Big/ \Delta[D]_{50\%}.m_{D-N(average)}$. Minimum error of 0.1 reported on Φ.

[c] Propagated error was 0.1 M for R20A and L35A and 0.2 M for L17A.

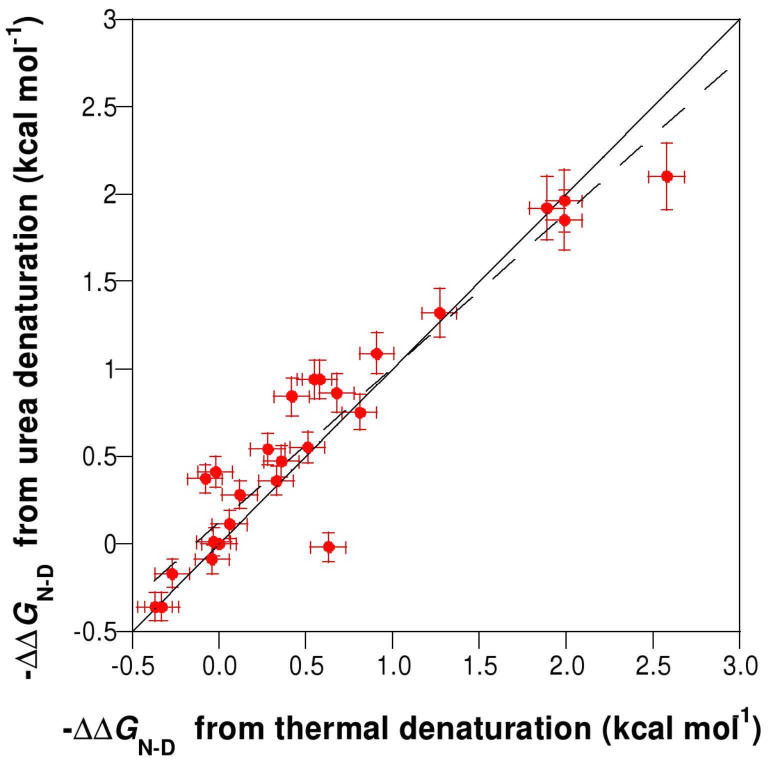

Supplementary figure S 1: Equilibrium stability of SAP mutants by chemical and thermal denaturation. Lines show regression fit to the data (dashed line) and y = x (solid line). Error bars show error from Table I (chemical denaturation) and fixed error of 0.1 kcal mol^{-1} (thermal denaturation).

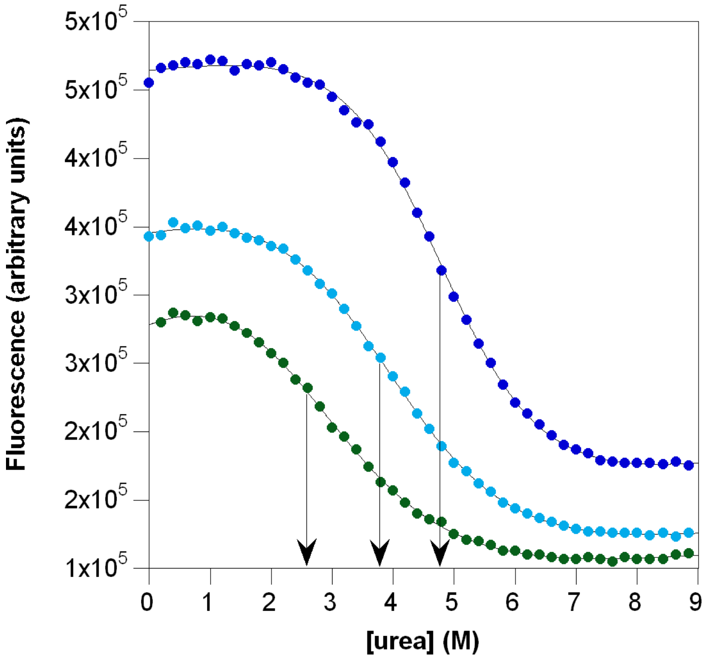

Supplementary figure S 2: Equilibrium chemical denaturation of SAP L31W. Urea denaturation at 283 K (dark blue), 293 K (light blue) and 303 K (green). Arrows indicate $[D]_{50\%}$ determined by curve fit (4.8 M at 283 K; 3.7 M at 293 K; 2.6 M at 303 K).
